# Controlling cost escalation of healthcare: making universal health coverage sustainable in China

**DOI:** 10.1186/1471-2458-12-S1-S8

**Published:** 2012-06-22

**Authors:** Shenglan Tang, Jingjing Tao, Henk Bekedam

**Affiliations:** 1 Duke Global Health Institute, Duke University, Durham, NC, USA; 2 School of Medicine, Shanghai Jiaotong University, Shanghai, China; 3 Western and Pacific Regional Office, WHO, Manila, Philippines

## Abstract

An increasingly number of low- and middle-income countries have developed and implemented a national policy towards universal coverage of healthcare for their citizens over the past decade. Among them is China which has expanded its population coverage by health insurance from around 29.7% in 2003 to over 90% at the end of 2010. While both central and local governments in China have significantly increased financial inputs into the two newly established health insurance schemes: new cooperative medical scheme (NCMS) for the rural population, and urban resident basic health insurance (URBMI), the cost of healthcare in China has also been rising rapidly at the annual rate of 17.0%% over the period of the past two decades years. The total health expenditure increased from 74.7 billion Chinese yuan in 1990 to 1998 billion Chinese yuan in 2010, while average health expenditure per capital reached the level of 1490.1 Chinese yuan per person in 2010, rising from 65.4 Chinese yuan per person in 1990. The repaid increased population coverage by government supported health insurance schemes has stimulated a rising use of healthcare, and thus given rise to more pressure on cost control in China.

There are many effective measures of supply-side and demand-side cost control in healthcare available. Over the past three decades China had introduced many measures to control demand for health care, via a series of co-payment mechanisms. The paper introduces and discusses new initiatives and measures employed to control cost escalation of healthcare in China, including alternative provider payment methods, reforming drug procurement systems, and strengthening the application of standard clinical paths in treating patients at hospitals, and analyses the impacts of these initiatives and measures. The paper finally proposes ways forward to make universal health coverage in China more sustainable.

## Introduction

An increasingly number of low- and middle-income countries have developed and implemented a national strategy and policy towards universal coverage of healthcare for their citizens over the past decade [1,2]. In so doing, national governments, employers and individual citizens have increased their investments on health and health care, aiming to increase population coverage, improve access to quality health care, and reduce financial risks for patients in seeking care. One challenge facing many countries is that people are living longer than they used to, owing to improved living standard, advanced health technology, better education, etc. In addition, high-technologies developed to advance healthcare in recent decades have also resulted in people’s higher expectations on health care. In the meantime, people are less active physically nowadays than they used to be, due to changes in the nature of working and life styles, resulting in more non-communicable chronically diseases (NCD). All these have been leading to the increasing needs of healthcare. Implementing universal health coverage, through either social health insurance, or national health services, would have to increase potential demands for healthcare, and thus the cost of healthcare may be rising more rapidly than the economic growth seen in many countries. That has led governments and employers, as well as individuals, to be more concerned about affordability to pay for health care. Therefore, controlling cost escalation of healthcare is always one of great challenges in scaling up the implementation of universal healthcare in these countries. "Value for money" is one of important topics in discussing and debating how countries can make universal health coverage more sustainable.

**Figure 1:**
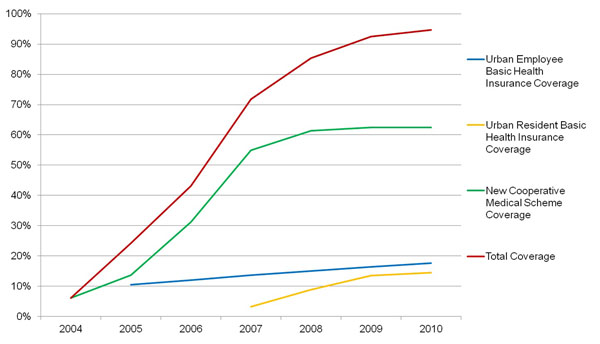
Changing tendency of population covered by the health insurance from the year of 2004 to 2010.

Since early 2000s the Government of China has seriously considered its social development after its rapid economic growth in 1980s and 90s. President Hu Jingtao and Premier Wen Jiabao have paid more attentions to the welfare of the Chinese population than the previous political leaderships which had seen the rapid growth of gross domestic products (GDP) as the top priority in China. Like many other developing countries, main sources of finance for healthcare in China come from central and local government funding, employers’ contribution to urban employee health insurance scheme, and individuals’ out of pocket payments including premium payment, co-insurance payment, etc. In late 2002, the Government of China decided to start to re-establish its rural health insurance scheme, named New Rural Medical Cooperative Scheme (NCMS), with financial supports from both central and local governments [3]. Central and local governments allocated RMB 20 yuan per rural resident in 2003 to support the NCMS, while individuals were required to contribute RMB 10 yuan/person. The financial support from governments and individual premium has now reached a level of at least RMB230 yuan per person in 2011, of which government subsidies accounted for around 80%. All the Chinese rural residents are eligible to enrol the scheme. Building upon what the Chinese government has done in the rural areas, the government in 2007 decided to launch urban resident basic health insurance (URBHI), trying to cover those urban population who are not covered by urban employee basic health insurance (UEBHI). Over the past decade, UEBHI, a social health insurance scheme jointly funded by employers and employees, has also been strengthened, covering more employees from public and private sectors than ever. By 2010, NCMS has now covered 834 million rural residents, while urban employee BMI and urban resident health insurance covered 429 million urban population by the end of 2010, i.e. over 90% of the Chinese population have now got health insurance coverage. Figure 1 would display the changing tendency of population covered by the health insurance from the year of 2004 to 2010.

As a result of this new development, access to healthcare in China has to some extent been improved in recent years. The cost of healthcare in China has been rising rapidly at the annual rate of 17.0% over the period of the past two decades. The total health expenditure increased from RMB 74.7 billion yuan in 1990 to 1,998 billion yuan in 2010 [4]. Average expenditure per hospital admission and per outpatient visit has increased at an annual rate that was by far much quicker than the average rate of GDP growth. Such a rapid rise may be partly attributed by meeting unmet needs of healthcare for the vulnerable groups, and partly owing to moral hazards associated with both demand for, and supply of, healthcare in China. A rapid increase of household income level in recent years may have also played a role in demand for higher quality healthcare in China. The Governments of China at national and local levels has recognized the issue of rapid cost escalation of healthcare, and many policy instruments and reforms have been implemented. These reforms, often through piloting studies, include: improved regulation of drug prices, development of new essential drug lists, using alternative provider payment methods, improving the procurements of drugs and other health technologies, and regulating the uses of high technologies. The main purposes of this paper are to present firstly the cost escalation of healthcare and its influencing factors in China, and then introduce and discuss initiatives employed to control cost escalation of healthcare in China, and analyse its pros and cons of such strategies. The paper ends with discussion on how China can learn from other countries to make its universal health coverage more sustainable.

## Healthcare cost escalation and its influencing factors

**Figure 2:**
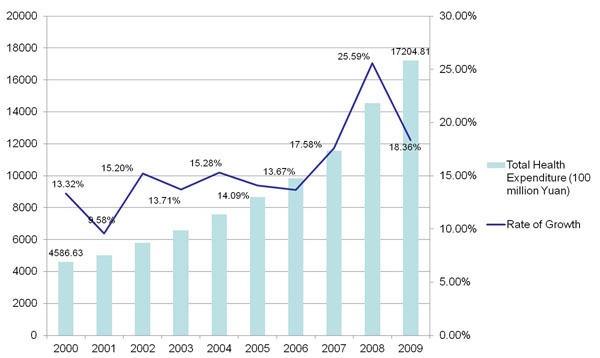
Cost escalation of healthcare since China enters the 21^st^ century in terms of total health expenditure and growth rate.

The cost escalation of healthcare has been one of hot topics in reforming national health systems for many decades. It has been also centred in the discussion and debates of China's health care system reform over the past three decades [5]. According to the national health account studies, the increase rate of health care costs in China has also been by far faster than the rate of economic growth over the period [6]. The average growth rate of healthcare over the past decade has been slowed down, but still at 15%. Figure 2 would demonstrate the cost escalation of healthcare since China enters the 21^st^ century, from two perspectives of total health expenditure and growth rate. The total health expenditures are paid for from three categories: government health expenditure, social health expenditure, and out of pocket expenditure. Over the past decade, the shares of the government health expenditure and social health expenditure have, respectively, risen from 15.5% and 25.6% in 2000, to 27.2% and 34.6% in 2009 (Table 1). As many studies reported, the government funding appropriated to the Chinese health facilities (e.g. public hospitals, primary health care centres, etc.) has relatively declined, since the early 1980s. A vast majority of Chinese health facilities have had to rely on service fees and profits from drug sale to generate revenues to cover their operational costs [7]. Worse is that the income of many Chinese health professionals, and other health staff welfare programmes, are linked to the extents of their abilities to generate revenues for their institutions [5].

**Table 1:** Who have paid for healthcare in China

Year	Government health expenditure		Social health expenditure		Out-of-pocket health expenditure	
	
	Level (100 million Yuan)	As percentage of health expenditure	Level (100 million Yuan)	As percentage of health expenditure	Level (100 million Yuan)	As percentage of health expenditure
2000	710	15	1,172	26	2,705	59
2001	801	16	1,211	24	3,014	60
2002	909	16	1,539	27	3,342	58
2003	1,117	17	1,789	27	3,679	56
2004	1,294	17	2,225	29	4,071	54
2005	1,553	18	2,586	30	4,521	52
2006	1,779	18	3,211	33	4,854	49
2007	2,582	22	3,894	34	5,099	40
2008	3,594	25	5,066	35	5,876	40
2009	4,686	27	5,948	35	6,571	38

Source of data: The Chinese Ministry of Health: *China Health Statistics Yearbook of 2010*. Beijing: 2010

Fee-for-service (FFS) has for many decades been used in China to pay health providers for the services provided, while government health budget allocated to public-owned health facilities, albeit being less important until recently, has been another main provider payment method. There are over 4,000 fee items in the FFS in China. Under such a circumstance, FFS has provided the doctors and hospitals in China with strong financial incentives for over provision of expensive health services. Total health expenditure is determined by prices of services and goods (e.g. drug prices), and volume of service use. The prices of services are often associated with input costs, nature of service providers (e.g. profit vs. non-profit), and other factors, while the rates of service use are associated with health insurance, individual income level, perceived health needs, structure of population, etc. As pointed out by the World Bank and other studies, FFS financial incentives for health service providers, together with weak regulation of health service providers, have led to over-use and unnecessary treatments and drug prescriptions. The national health services survey undertaken in 2008 reported that unnecessary hospital admission might be as high as 30% nationally [8]. This could be partially contributed to a significant rise in numbers of outpatient visits and inpatient hospital admissions from 2002 to 2010, as shown in Table 2. While the number of Chinese population has not changed much over the period, the number of outpatient visits and hospital admissions increased from 21.5 trillions and 59 millions in 2002 to 58.4 trillions and 141.7 millions, respectively. It is reasonably believed that the rapid expanded population coverage by the health insurance schemes, hugely subsidized by the central and local governments in China, did play a role in such an increase of health service uses. Moral hazards have inevitably been associated with some unnecessary use of limited health resources, as the first author of the paper witnessed that some township health centres admitted patients with minor illnesses for hospitalization. Such a rapid increase of healthcare use has a huge implication for cost escalation of health care in China.

**Table 2:** Number of outpatient visits and inpatient admissions by level of health institution in China (2002-2010)

Year	Total population (100,000,000)	Total		Hospitals		Community health centres		Township health centres	
		
		Total OP visits (100,000,000)	Total IP admissions (10,000)	OP (100,000,000)	IP (10,000)	OP (100,000,000)	IP (10,000)	OP (100,000,000)	IP (10,000)
2002	12.85	21.45	5,991	12.43	3,997		11	7.1	1,625
2003	12.92	20.96	6,092	12.13	4,159	0.38	10	6.91	1,608
2004	13.00	22.03	6,669	13.05	4,668	0.46	15	7.03	1,621
2005	13.08	23.05	7,184	13.87	5,108	0.59	27	6.79	1,622
2006	13.14	24.47	7,906	14.71	5,562	0.83	44	7.25	1,858
2007	13.21	33.32	9,827	16.38	6,487	2.26	107	7.59	2,662
2008	13.28	35.3	11,483	17.81	7,392	2.57	141	8.62	3,355
2009	13.35	54.88	13,256	19.22	8,488	3.77	225	8.77	3,808
2010	13.40	58.4	14,174	20.4	9,524				

Source of Data: The Chinese Ministry of Health: *China Health Statistics Yearbook of 2010*. Beijing: 2010

As described in the introduction section, three mainstream health insurance schemes in China have been developed rapidly in recent years, covering a vast majority of the Chinese population. URBHI and NCMS are jointly funded by national/local governments and individuals, while UEBHI is jointly supported by employers and employees. The resource pooling and risk-sharing level varies from place to place, but most of the health insurance schemes in China are at county/district level, and some at city/municipal level. The management centres of these schemes have been responsible to develop service benefit packages based on the guidelines issued from central government. They have also started to play a modest role in cost control of healthcare, mainly in the area of developing alternative payment methods to health service providers, although FFS is still the most common method used in China, as said above.

## Cost control initiatives in China and their impacts

Containing the cost of health care has become one of main objectives of virtually all national health systems around the world - both high-income and low- and middle-income countries. Over the past decades, the high-income countries have developed many measures to tackle the cost escalation of health care. These measures target either demand side or supply side. Among the measures targeting the demand side are co-payments (e.g. deductable and co-insurance payments and ceiling), limited benefit packages, etc, while those targeting supply side include the use of different provider payment methods, (e.g. capitation, DRG payment, capped global budget), regulation of drug and service prices, controlling the use of high-technologies, etc. All these measures do have impacts on cost control. However, there are also implications for equity in access to healthcare, and efficiency of service provision, as well as quality of care. Therefore a balance in using the combination of the measures needs to be found.

In tackling the cost escalation of health care in 1980s and 90s, there seems a trend in China that the measures targeting service users were more easily introduced and implemented than the measures targeting service suppliers. For example, in reforming UEBHI, high deductable and co-insurance payment, as well as reimbursement ceiling was introduced as early as in late 1980s. So were the limited service benefit packages which defined which services or drugs are reimbursable or not. In the meanwhile, although great efforts were made to introduce alternative provider payment methods to FFS, it was less successful, particularly in a large scale. This may owe largely to the fact that the service users have weak political voices than the service providers and other key stakeholders (such as national and international pharmaceutical manufactures) in China. Furthermore, even if the government did take some cost controlling measures targeting service providers and medical suppliers, the effects were often comprised because of coping strategies developed by the supply side. For example, the National Reform and Development Commission, a government authority responsible for pricing regulation, among others, has since the 1990s regulated (mainly reduced) the prices of drugs over 20 times. Once such policies and measures were implemented, many pharmaceutical manufactures stopped the production of drugs that have to be priced lower than what they expected, while doctors and hospitals in China were less likely to use these drugs, as such policies and measures would affect profit making and revenue generation [9]. Both central and local governments in China have been fully aware of the problems and challenges in addressing the cost escalation of healthcare. Many policy initiatives have been put in place to use limited resources more efficiently. This section would introduce several emerging initiatives of cost control targeting the supply side of healthcare markets in China and examine their effects.

### Using mixed provider payment methods

Provider payment methods consist of retrospective (e.g. FFS, case-based/DRG, and unit flat rate), prospective (e.g. salary, capitation, and global budget), and mixed payment methods. Different provider payment methods have different advantages and disadvantages in terms of cost and quality of healthcare. An increasing number of countries are in favour of using mixed payment methods to control rapid escalation of healthcare cost. Using alternative provider payment methods to FFS in China has always been a great challenge. Over the years, NCMS, UEBHI and URBHI in some cities/counties of China have been trying to use case-based payment or flat unit rates to pay outpatient visits and hospital admissions. In some cities, global budget was used to pay for defined number of hospital admissions. Case-based payment system is one of main national initiatives developed to tackle the cost escalation of healthcare, among other issues. In 2004, the Ministry of Health, China endorsed a policy that supports experiments in the application of case-based payment system in China. Since then an increasing number of provincial health authorities have decided to pilot this provider payment method, although such a payment reform had been seen in several cities of China before. A common problem with case-based payments is often the limited number of diseases covered. If too few diseases are covered, the overall impact on cost control at a hospital level is most modest. Hence, many health insurance schemes in different cities/counties of China have adopted flat unit rates for an outpatient visit or/and inpatient admission at different level of health service providers. Such a payment can simplify the management procedures and would have an overall impact on the total health expenditure. Global budget has also increasingly been used to ensure that the health insurance funds would not get into deficits. We have selected three cities in China: Shanghai in the east-coast), Shenzhen in the southern, and Mudanjiang in the north-east to look at what these cities have done in reforming their provider payment methods in order to tackle the rapid cost escalation of health care, among others.

Table 3 introduces main provider payment methods used in the three cities, and other key measures adopted to complement the effort of cost control. It also presents their impacts on overall cost control of health care and the balance of health insurance funds. As we can see, all the three cities have used more than one provider payment method to pay for outpatient and inpatient services. One key message from the table is that, except a few situations, all the three cities have tried to use alternative methods to FFS. FFS, as the most popular method used over the past decades, has been associated with the rapid cost escalation of health care in China and other countries. The results show that either case-based payment, or using flat unit rate to pay for OP and IP services, or global budget, have produced positive impacts on cost control of health care in the three cities. Other measures on rational use of drugs and high-tech, as well as strengthening service referring system might have also been attributed to slow down of cost escalation in these cities. While these alternative provider payment methods did produce the results that the Chinese policy-makers and health insurance fund managers wanted to see, there have also been problems and challenges reported in these cities. In Shanghai, studies found that doctors in the CHCs were more likely to refer outpatients to higher level hospitals in order to reduce their own costs. In Shenzhen, the hospitals and doctors failed to provide necessary diagnostic tests for the patients, while doctors in Mudanjiang encouraged their patients to come back to see them after 3-4 days immediately, as a new case. As Shanghai uses the global budget to pay for hospital admissions, many patients, who needed hospitalization in the period of approaching fiscal years, were often delayed in admission to hospital care. In Shenzhen and Mudanjiang where case-based payment was used, hospitals and doctors tended to diagnose their patients as more severe cases as they actually were, in order to claim higher payment rates, as found in other countries, or some hospitals let their inpatients discharged from hospital care and then re-admitted them. As a result, studies undertaken in these three cities reported less patients' satisfaction with the services provided, and the relationship between service users and providers has become tense. In addition, many health insurance fund agencies felt lack of adequate technical capacity to expand the application of the case-based payment to cover more diseases.

**Table 3:** Provider payment methods and their impacts on cost control of health care in selected cities of China

		Shanghai	Shenzhen	Mudanjiang
Provider payment methods		• Global budget as main method; • Case-based payment for specific services [10].	• Fixed unit rate for inpatient services; • Case-based payment for specific services [10].	• Case-based payment as main method; • Fixed unit rate and FFS for specific services [10].
	OP services/ CHCs	Global budget	FFS for outpatient services	Fixed unit rate for OP visits.
	IP admission	Global budget, while case-based payment used for selected diseases	A fixed unit rate payment for hospital admissions	Case-based payment for IP admission, with capped annual ceiling.
	Special cases/services	Flat daily rate payment for inpatients with mental health problems	Case-based payment for normal baby deliveries	FFS for specific services as defined.

Other cost control measures	Rational use of drugs and high tech	Drug expenditure as % of total health revenue of designated hospitals by health insurance scheme should reduce year by year; Regulate the services and prices of high technology related services [11].	A monitoring and evaluation of prescribed drugs introduced in all the public hospitals. Expenditure of antibiotics should not be more than 20% of the total drug expenditures.	Each prescription provides patients with drugs for only up to 3 days [14].
	Service delivery management	Strengthening management of service referring system.	Defining the ratio of outpatient visits to IP admission to control induced hospital admissions [13].	Level of case-based payment or unit rate differs between different levels of hospitals [15].
Impacts	Cost escalation of health care	The annual increase rate of health care expenditure for main health insurance schemes has been around 11.7% a level similar to the annual GDP growth rate in Shanghai [12].	Average health expenditure per the insured has been maintained at a stable level (e.g., RBM 646.2 yuan in 2003, RBM 587.3 yuan in 2004)	The increases in OP and IP expenses were slower than the increase of average GDP per capita. The 2007 statistical data show that the average expenditure per hospital admission in the city was 28% lower than the national average [16].
	Health insurance funds	A balanced situation maintained in terms of incomes and expenditures.	The health insurance fund maintains a modest surplus annually	The health insurance fund has a modest surplus.

### Separating revenue and expenditure system

In recent years, both central and local governments in China have significantly increased its health budgets to urban community health centres/stations, and township health centres in the rural areas. These facilities are expected to provide their communities with public health interventions and essential clinical care. Prior to the new health system reform, these health facilities received government health budgets which only covered a small proportion of total operational costs. Hence, they had to generate revenue from user fees to cover financial gaps. Over the past decade, reforming government health budget payment has been under way, especially in the primary health care providers. "Separating revenue and expenditure system (SRES)" is one of most popular reforms. Strictly speaking, this should also lie in the scope of the provider payment methods, as discussed in the above section. However, we introduces and discusses it separately, as it addresses an unique issue of removing perverse incentives to generate revenues through over provision of services, i.e. delinking incomes from revenue generation in Chinese health facilities. Under SRES, all the revenues, including incomes from service charges and drug sale, are paid into a special government account, while each service provider will be paid for by the county/district bureau of finance, according to agreed health budgets [17]. Such an initiative has been seen in Beijing, Chengdu, Chongqing, Shanghai, Tianjin, and Yinchuan, and other Chinese cities (Table 4).

**Table 4:** Impacts of SERS on health care in selected pilot areas of China

Impacts	Beijing	Chengdu	Hangzhou
Revenues/ expenditures of CHCs	Proportion of drug expenditure and service charges declined as % of the total health expenditure of CHC; CHCs might not be to receive the payment from governments timely to cover the expenditure [12].	District/county governments increased funding to CHCs; Ave expenditure per outpatient visit declined; CHCs sometimes did not receive payments from governments or social health insurance timely	District/county governments increased financial inputs under SRES; Ave expenditure of outpatient visit declined; CHCs might not receive the payments from government timely [17,31].
Quantity of services provided	The quantity of outpatient visits and public health services provided in CHCs increased significantly; No changes in home visits [14].	The quantity of outpatient visits increased significantly; While more NCD patients have been effectively managed, many NCD patients bypassed CHCs to seek tertiary care	The use of CHCs increased; CHCs provided more public health services related to NCDs control [18].
Quality of care	Patients' satisfaction with outpatient services increased, as more patients chose CHC as the first contact with professional care; No changes in the management of NCDs [15].	Patients' satisfaction with the services increased, resulting in high use rate; Lack of qualified general practitioners prevented further increase of quality of care	Overall satisfaction with the CHC services increased significantly; Lack of qualified general practitioners resulted in slow development of CHCs [19].
Perceptions of community health workers (CHWs)	SERS can ensure the income of CHWs, and reduce unnecessary treatments that used to produce profits for CHCs, making healthcare at community level more affordable; SERS does not provide CHWs with financial incentive to work hard [16].	Most CHWs were satisfied with the reform, while others were less keen to provide public health services, as defined in the SERS.	Increased workload, particularly related to NCD control, at CHCs may not be sustainable; While salaries of CHWs are secured, the income level did not match the increased level of workloads. Many CHWs were not satisfied with their income levels after the reform [18,31].

#### SRES in urban community health centers (CHCs) in Beijing, Chengdu and Hangzhou

Since 2007 all the CHCs in 18 districts/counties in Beijing have implemented SRES [18]. All the revenues from these centres are paid into a special account held in the district/county bureau of finance. All the expenditures of the centres are paid for according to the agreed budget lines including personnel salaries/subsidies, drugs, other medical supplies, etc. The income of health workers in these centres are no longer linked to the level of revenues generated from service charges and/or drug profit [19].

In Chengdu, Sichuan Province, the principles for implementing SRES are similar to what Beijing has done. However, it emphasized that, while recurrent expenditures should be paid for by the district/county governments, the infrastructure/capital investment for the CHCS should be jointly financed by district/county and Chengdu municipal government [20]. In addition, the district/county governments should sign an annual contract with each CHC defining what public health and essential clinical services are purchased with agreed budgets. Like Chengdu, Hangzhou applied the same principles, as Beijing has done, in implementing SRES.

#### Impacts of SRES initiative

Table 5 presents the impacts of SRES initiative on four aspects: 1) changes in health expenditure, 2) changes in quantity of services provided; 3) quality of care, and 4) perceptions of community health workers. These results were derived from a review of Chinese literature which published assessment studies in selected cities where SRES was piloted. It is apparently clear from the table that there have been both positive and negative effects emanating from the initiative in these pilot areas of China.

**Table 5:** Impacts of SERS on health care in selected pilot areas of China

Impacts	Beijing	Chengdu	Hangzhou
Revenues/ expenditures of CHCs	Proportion of drug expenditure and service charges declined as % of the total health expenditure of CHC; CHCs might not be to receive the payment from governments timely to cover the expenditure [19].	District/county governments increased funding to CHCs Ave expenditure per outpatient visit declined CHCs sometimes did not receive payments from governments or social health insurance timely	District/county governments increased financial inputs under SRES; Ave expenditure of outpatient visit declined; CHCs might not receive the payments from government timely [24,27].
Quantity of services provided	The quantity of outpatient visits and public health services provided in CHCs increased significantly; No changes in home visits [21].	The quantity of outpatient visits increased significantly While more NCD patients have been effectively managed, many NCD patients bypassed CHCs to seek tertiary care	The use of CHCs increased; CHCs provided more public health services related to NCDs control [25].
Quality of care	Patients' satisfaction with outpatient services increased, as more patients chose CHC as the first contact with professional care; No changes in the management of NCDs [22].	Patients' satisfaction with the services increased, resulting in high use rate. Lack of qualified general practitioners prevented further increase of quality of care	Overall satisfaction with the CHC services increased significantly; Lack of qualified general practitioners resulted in slow development of CHCs [26].
Perceptions of community health workers (CHWs)	SERS can ensure the income of CHWs, and reduce unnecessary treatments that used to produce profits for CHCs, making healthcare at community level more affordable; SERS does not provide CHWs with financial incentive to work hard [23].	Most CHWs were satisfied with the reform, while others were less keen to provide public health services, as defined in the SERS.	Increased workload, particularly related to NCD control, at CHCs may not be sustainable; While salaries of CHWs are secured, the income level did not match the increased level of workloads. Many CHWs were not satisfied with their income levels after the reform [24,27].

Overall speaking, the perverse financial incentive previously given to the service providers has now been removed. As a result of that, the over-provision of services and over-prescriptions in these health centres have been rationalized, which has led to improved economic efficiency and increased patients' satisfaction with the services provided. The provision of public health interventions/services has also been improved, as the initiative has a strong mandate to strengthen public health at the community level. Many community health workers have now felt that their incomes have been secured, but the health workers who used to get high income and/or bonus payment, linked to their service charges, have seen the decline of their income levels, while their workloads, particularly related to public health services, have increased [23,27,28]. Another concern reported is that many CHCs could not receive the transfer of the funding timely from district/county governments which could affect the operation of service provisions [19,27].

### Reforming management of pharmaceutical distribution/procurement systems

The national health account studies show that China spent some 5.13% of GDP on health care in 2009, of which 42% of the expenditure was on pharmaceuticals [23]. The proportion was one of the highest shares of pharmaceutical expenditure in total health expenditure in the world, compared to an average of around 15% in the OECD countries [29]. One problem associated with excessive use of drug lies in the distribution chain of pharmaceuticals in China. Since the economic reform launched in the late 1970s, both the drug distributors and manufacturers have been allowed to sell drug directly to hospitals and pharmacies. In other words, each of the 4,600 pharmaceutical manufacturers in China can also act as distributors apart from those 12,000 wholesales [9]. They often used proactive ways, legally or illegally (e.g. kick back to hospital and pharmacy managers) to promote the sale of their products. In order to reduce the drug expenditure as percentage of total health expenditure and unnecessary use of expensive drugs including antibiotics, the Government of China, with the support from WHO, has taken a series of actions to improve value for money. One major reform was to revise the list of national essential drugs since 2004. The number of Western medicines and manufactured Chinese drugs reduced from over 2,000 in the 1990s to only 307 in 2009. In addition, primary health care facilities including urban health service centres/stations and township health centres/village health stations are only allowed to use essential drugs in many parts of China. Policies on encouraging rational use of drugs have also been put in place by local health authorities over the past decade. NDRC and provincial bureaux of pricing have strengthened the regulations of drugs. Often under the SRES, the health facilities are no longer allowed to sell drugs with 15-20% mark-up rates (so-called zero-profit for drug sale in China), which they normally did before the reform. In addition, the systems of drug distribution and procurement have also been reformed over the past decade. Many local health authorities have developed different measures aimed to reduce the cost of drug procurements. Below are several main procurement initiatives being used in China [30].

#### 1. Collective bidding

Under this system, all the public hospitals in a province or city/prefecture use their collective bargaining power to purchase required drugs for up to three years. The local health authorities first prepare the purchasing plan defining clearly what products and quantities of these products, models of deliveries, and procedures/rules and then publish their bidding documents. Any qualified pharmaceutical manufacture or supplier can join the bidding with indicative price for each product. The health authority will then review these bids and select on to sign a contract. In order to be fair, almost all the review processes follow the principle of blinding evaluation, i.e. the reviewers will not know which bid from which company. Quality, prices, company's reputation, and delivery services, are the key indicators for the review and selection of the bids.

#### 2. Internet-based price bidding

Hospitals and other health service providers publish the needs of pharmaceutical products and their quantities at the internet-based pharmaceutical procurement information platform. They also indicate the max price of each product they would be able to pay. Pharmaceutical manufactures or suppliers, who need first to register with the platform with relevant information of their companies, can make an offer to a particular tender. Often there are three rounds of bidding, and those offering relative higher prices would be out of the bidding process in the earlier rounds. Such a bidding process enables these pharmaceutical suppliers to be more competitive. Pricing is the most important factor considered, provided that the quality of product meets reasonable standards.

#### 3. Commissioned procurements

Commissioned procurements mean that one health authority or one hospital contracts one pharmaceutical distribution firm to take a full responsibility for drug purchasing, supply and storage. Such an arrangement was often carried under the auspice of the local health authority (e.g. county or distribute bureau of health), A number of public hospitals and other health facilities selected a pharmaceutical distributor through a bidding process. Once the distributor has been contracted, it would try to negotiate and purchase drugs, according to the needs of these health service providers, from different pharmaceutical manufactures or wholesales. The financial profits made will be split between the health service providers and the contracted distributor, based on the agreed terms. For example, it is normal for Chinese hospitals to have its own pharmacy within a hospital promise. Recently, Nanjing Municipal Bureau of Health in Jiangsu Province decided to contract one large pharmaceutical distributor to operate the pharmacies for all the public hospitals.

There are pros and cons of each model introduced above. The collective bidding system considers a number of factors, such as quality, price, product delivery, company reputation and so on, while the internet-based price bidding puts the factor of price as the most important indicator, provided that all the products meet the standard defined by State Food and Drug Administration (SFDA) in China. However, the collective bidding system uses a committee to review the bidding documents, while the internet-based price bidding uses IT software to select the winner. The former may be influenced by invested stakeholders, while the latter is probably more transparent. The commissioned procurements system has adopted a very different approach that the health service providers decentralize its decision powers to contracted pharmaceutical distributor. The impact of such a system needs to be assessed. Table 6 presents the results on average expenses of 13 anti-hypotension drugs (Daily Drug Dosage - DDD) from three cities, one of which (Guangzhou) used the collective bidding to purchase these drugs. As we see from the table, the prices for seven out of 13 drugs from Guangzhou were significantly cheaper than that of other two cities. Only two drugs were more expensive in Guangzhou than in Tianjin and Shanghai, while four in Guangzhou were between the two prices seen in the two cities. Overall speaking, the average DDD in Guangzhou was the lowest - RBM 3.80 yuan, compared to RMB 4.64 yuan in Shanghai, and RMB 5.12 yuan in Tianjin.

**Table 6:** The minimum DDD cost of anti-hypertension medicines in Guangzhou, Tianjin and Shanghai

No.	Generic name	Average minimum DDD cost		
		
		Liwan district, Guangzhou	Hebei district, Tianjin	Luwan district, Shanghai
1	Losartan	7.65	7.19	7.13
2	Captopril	0.04	3.33	0.39
3	Nitrendipine	0.09	0.05	0.13
4	Enalapril	1.38	1.99	1.61
5	Indapamide	0.72	0.94	0.51
6	Amlodipine	3.27	5.91	4.75
7	Levamlodipine	8.62	7.09	5.89
8	Benazepril	3.41	2.92	3.87
9	Perindopril	3.87	4.03	4.05
10	Fosinopril	5.58	5.55	3.78
11	Valsartan	4.48	5.47	5.24
12	Irbesartan	3.53	3.66	5.74
13	Felodipine	3.14	6.69	5.00
Average		3.80	5.12	4.64

Source of Data: Ministry of Health, China: *Report on pharmaceutical purchasing and utilization in primary health institution in China*. Beijing; 2009.

### Implementing standard clinical treatment path

Over-provision of services and over-prescription of drugs have been common in most Chinese hospitals, as discussed above. Clinical governance has been one of main challenges in reforming public hospitals in China. With financial support from the World Bank and Department for International Development, UK, and technical support from National Institute of Clinical Excellence (NICE), UK, the Ministry of Health, China has selected six district/counties in China to pilot the experiment of implementing standard clinical treatment path, aiming to improve the quality of healthcare, and control cost escalation. QJ district hospital in Chongqing City was chosen as an intervention hospital, while other two district/county hospitals in Chongqing City (WL and RC) as control hospitals. XH first district hospital in Shaanxi Province was chosen as intervention groups, while other two district/county hospitals in Shaanxi Province as control group. Experts from NICE worked with Chinese clinical experts and health economics to develop protocols of standard clinical treatment path for the selected diseases, and estimated the cost of the treatments, based on evidences available internationally and in China. The Chongqing study selected ten common diseases, while the Shaanxi study had 12 common diseases. Appropriate training workshops were organized by the Chinese experts involved in the standard/guideline development, using approved protocols of clinical treatment paths. In the meantime, the monitoring and evaluation systems have been established in the six district/county hospitals for data collection and analysis. The results presented below were based on the data collected from October 2010 to May 2011, totalling eight months [31].

The impacts of such an initiative were assessed from three aspects: quality of care, cost of health care, and technical efficiency of service provision.

1. Quality of care - indicators used to assess the quality of care, including mainly cure rates and use of drugs including antibiotics. Due to lack of clear yardsticks and definitions on what means "cured" or "almost cured, the comparison of quality of care between pre-intervention and post-intervention, and between the intervention group and control group was not meaningful. However, the use of drugs and particularly antibiotics declined significantly in the intervention group than in the control group. In addition, the two hospitals in the intervention groups started to use cheap, but effective, antibiotics again, such as penicillin, as these antibiotics are of first choices in the clinical treatment paths.

2. Cost of healthcare - average expenditure of hospital admission for over 50% of selected diseases declined significantly after the implementation of the clinical treatment paths in the intervention groups, while this did not change much in the control group. As for those diseases whose average expenditure of hospital admissions being increased, an in-depth analysis found that in the most cases the expenditure related to the use of diagnosis and drugs came down, while the cost of other medical supplies went up, due to high inflation witnessed in China over the study period. It is clear that irrational cost escalation of healthcare has to some extent been control.

3. Efficiency of service provision - indicators related to average length of hospital stay, average number of days prior to surgical operation were used to efficiency. The two comparative studies in Chongqing and Shaanxi showed declined in the two indicators. That means that the use of available health resources in the two intervention hospitals has increased.

Case-based payment was used to pay for the treatment of ten and 12 diseases, respectively in QJ district hospital of Chongqing, and XH district hospital of Shaanxi. It implies that reduction of average expenditure of hospital admission did not reduce the generation of revenue for these two hospitals and thus would affect the financing of hospital operations per se.

## Way forward for China: what are next steps

Over the past decade China has tried hard to develop effective measures to control the rapid cost escalation of healthcare. This is especially important, as the Government of China has been implementing the strategy of universal health coverage and increased significantly financial input into the health systems strengthening. Experiences and lessons from many OECD countries over the past half century would be useful and valuable to the on-going reform of China's health care system. They would help China to look for more effective and evidence-based measures to tackle the issues, making its universal health coverage more sustainable, though considerations on different contexts, such as political, economic and cultural factors, are required in the exercise of "know-how".

Ensuring high quality and safety of health care is one precondition in discussing how containing health care cost. It is very encouraging to see that some Chinese health facilities, with the technical support from NICE in England, have adopted the standard clinical paths for the diagnosis and treatment of their patients. This would have to ensure the effectiveness of using both diagnostic tools and clinical interventions, and also avoid the provision of unnecessary services whose main purpose was to generate revenues for services providers, and which may do possible harms, rather than any good, to patients. In many OECD countries, medical associations, or health authorities are responsible for updates of the clinical guidelines and treatment standards, using available evidence. The practice of this kind was not common in China until recently. Ministry of Health of China have now recognized the importance of standard clinical guidelines and made great efforts in setting clinical guidelines and norms in recently years, particularly for primary health care facilities handling common diseases and health problems. Greater efforts should be made to expand the application of standard clinical paths in more Chinese hospitals. Furthermore, as presented in this paper, there is lack of sufficient evidence generated from these pilot studies to inform further development of policies and practices. Research on impact assessment, including monitoring and evaluation, need to be encouraged to provide robust evidence and sound knowledge aimed to improve quality of care in China.

It is critically important and essential to have clinical guidelines and standards that health service provider can apply in their practices. However, we all know that are not sufficient. Appropriate incentives must be developed to enable service providers to implement good practices of health services that are accessible and affordable to the people in need. As reported in this paper, many local health authorities in China have taken proactively roles in developing and implementing local initiatives aimed to provide health service providers with appropriate mechanisms and incentives for the delivery of health care services. SRES has become one of widely used mechanisms, particularly in financing of primary health care centres in urban cities of China. It seems that such a mechanism removes a perverse financial incentive to the over-prescription of diagnosis and drugs and delinks the income of community health workers with their revenue generation from service charges and drug prescriptions. As a consequence, rational prescription of diagnosis and drugs can be promoted to make essential healthcare more affordable. In addition, the fund for public health inventions can be protected from such an allocation of government health budgets. However, measures needs to be developed to ensure that the transfer of the government health budgets to these CHCs should be made in timely manner in order to operate community health centres in an effective and efficient way. Furthermore, how to develop innovative incentives (i.e. financial and professional) for community health workers is facing a new challenge.

As we know, there are no perfect provider payment methods exist. China has been in a right direction in reforming provider payment methods to tackle cost escalation of health care in recent years. Case-based payment method and flat unit rate payment have been increasingly used in China. Some cities, like Shanghai, have also started to use global budget to pay for health care. The implementation of case-based payment system in China must be done carefully, as only a number of diseases were often covered in such a system. The hospitals may shift the costs from the treatment of these selected diseases to the treatment of other health problems. In addition, as reported in this paper, there have also some concerns about the quality of services, under-treatment, and unnecessary patient referring seen in some hospitals in order to save the costs of care. That is not surprising. Our experiences also tell that service providers often developed their coping strategies to deal with any reforms or initiatives to regain their invested interests. It implies that the monitoring and evaluation of these reforms and their impacts on cost and quality of healthcare is critically important. And, the health authorities or health insurance management agencies should be well prepared to change policies or strategies every a couple of years. Furthermore, in order to achieve this, it is imperative to improve significantly technical capacities of the local health authorities and health insurance management agencies in China.

Drug expenditure as percentage of total health expenditure in China is substantial over the past three decades. In order to reduce the cost of drug expenditure, the management of pharmaceutical procurement system has been radically reformed in recent years, which has produced a positive impact on bringing the prices of many drugs down at the point of service delivery, as we have seen in Guizhou. What China should do more is that local health authorities (e.g. provincial or municipal levels) and/or health insurance fund management agencies at these levels ought to use their large collective bargaining power to get reasonably low prices of drugs, particularly for most generic ones, as Britain's NHS and other OECD countries has been doing. There is still a room for Chinese health authorities or health insurance schemes to do more in this area. Challenges facing the health sector in China include trade protections within different provinces, weak purchasing capacity of some health authorities and health insurance management agencies in less developed regions, and strong lobbying from international and local pharmaceutical manufacturers and wholesale agents. While it is imperative to improve the rational use of drug by developing and implementing appropriate incentives for both service users and providers, it is also equally important to ensure that these innovative pharmaceutical procurement systems, including those tendering practices aimed to low drug prices, would not jeopardize or discourage internal innovation in the Chinese pharmaceutical sector, and diminish enthusiasm of multi-national drug firms to participate in the China market. Overall speaking, the systems of drug distribution, procurement and use in China have been improved gradually.

China's health system reform is at a critical moment. Although both central and local governments have increased their funding for implementing universal health coverage over the past decade, and an increasing number of Chinese people have now enjoyed affordable essential healthcare, the task of cost control in the health sector is still formidably challenging. Without effective containment of health care cost, increased investments in health care would not be transferred to improved access to health care for Chinese people in need, and thus the goal of fully implementing universal health coverage by 2020 would have to be in jeopardy. More effective actions and measures on cost control are badly needed in China now than ever.

## List of abbreviations used

NCD: Non-communicable chronically diseases NCD; GDP: Gross domestic products; NCMS: New Rural Medical Cooperative Scheme; URBHI: Urban resident basic health insurance; UEBHI: Urban employee basic health insurance; FFS: Fee-for-service; SRES: Separating revenue and expenditure system; CHC: Community Health Centres; SFDA: State Food and Drug Administration; DDD: Daily Drug Dosage; NICE: National Institute of Clinical Excellence; OP: Outpatient; IP: Inpatient.

## Competing interests

The authors declare that they have no competing interests.
